# Fatal Orbital Cellulitis With Intracranial Abscess: A Case Report

**DOI:** 10.7759/cureus.42435

**Published:** 2023-07-25

**Authors:** Ru Jian Jonathan Teoh, Yin Peng Lai, Rohanah Alias

**Affiliations:** 1 Ophthalmology, Hospital Kuala Lumpur, Kuala Lumpur, MYS

**Keywords:** odontogenic, fatal infections, intracranial extension, intracranial abscess, orbital cellulitis

## Abstract

Orbital cellulitis is a sight- and life-threatening condition. Prompt diagnosis and immediate intervention are required. We report a case of fatal odontogenic orbital cellulitis complicated with intracranial abscesses in a 55-year-old gentleman. The patient presented with painful swelling of the left eye associated with reduced eye movement, blurry vision, and a headache. There was generalized periodontitis. Initial imaging was suggestive of left eye orbital cellulitis with intracranial abscess, and intensive systemic antibiotic therapy was initiated. After six weeks of antibiotic therapy, there was an improvement in ocular signs and symptoms. However, the patient developed signs of meningism with a persistent fever. Serial brain imaging demonstrated worsening intracranial abscesses. The patient died eight weeks after the initial onset of presentation. This case emphasized that brain abscesses could be a fatal complication of odontogenic orbital cellulitis. A high index of suspicion is important in diagnosing orbital cellulitis and its complications. Early consideration of surgical intervention is necessary in cases not responding to antibiotic therapy.

## Introduction

Orbital cellulitis is an infection that affects the tissues posterior to the orbital septum within the orbit. It can occur at any age but is more common in the paediatric population [1]. Orbital tissues could be infected via direct inoculation such as trauma or insect bite, the direct spread of infection from adjacent structures such as sinusitis and dacryocystitis, or haematogenous spread of infection from a distant source such as otitis media [2]. The commonest source of infection in all age groups is the paranasal sinuses, most commonly the ethmoid and maxillary sinuses [2,3]. Other predisposing factors include the spread of upper respiratory tract infections, ocular trauma, ocular surgery, iatrogenic and post-traumatic foreign bodies, dacryocystitis, and dental infections [2,3]. Orbital cellulitis could progress to sight-threatening and fatal complications if the infection extends to adjacent structures [3]. Ocular complications include exposure to keratopathy, corneal ulceration, uveitis, iridocyclitis, choroiditis, panophthalmitis, glaucoma, optic neuropathy, and retinal detachment [3]. Intracranial complications occur when the infection extends to the brain tissue, resulting in intracranial abscess, meningoencephalitis, and cavernous sinus thrombosis [3]. In this case report, we review a case of fatal orbital cellulitis with intracranial complications in an adult.

## Case presentation

A 55-year-old gentleman with underlying poorly controlled type 2 diabetes mellitus presented with a two-week history of painful left eye swelling and frontal headache. He reported difficulty in moving his left eye, which was associated with redness and blurry vision. He had toothache a week prior to the ocular symptoms. There was occasional nausea and intermittent low-grade fever.

**Figure 1:**
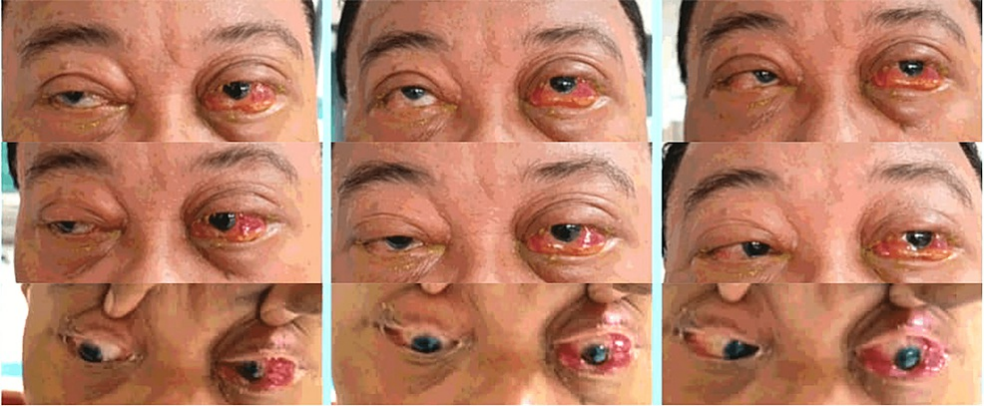
Initial presentation of the patient with 360-degree conjunctival chemosis of the left eye, with limited ocular movement at all gazes

On examination, the patient was septic-looking. The best corrected visual acuity was 6/60 in the left eye and 6/9 in the right eye with no relative afferent pupillary defect. The left upper eyelid was erythematous. There was 360-degree conjunctival chemosis with limited extraocular movement, i.e., -3 on horizontal gazes and -2 on other gazes (Figure 1). Intraocular pressure (IOP) of the left eye was 26. Otherwise, there was no abnormality found in the cornea, anterior chamber, and posterior segment of the left eye.

Biochemical parameters revealed raised inflammatory markers with C-reactive protein of 281 mg/L and raised white cell count (14.1 x 10^9^/L) with a neutrophil predominance. Blood culture and urine culture demonstrated no growth of microorganisms. The infective screening was unremarkable. Contrast-enhanced computed tomography (CECT) of the brain and orbit revealed a heterogeneously enhancing lesion at the left pre-septal and post-septal region involving the orbital tendon of the left lateral rectus, and enhancing subdural collection in the left frontal region measuring 0.3 cm x 0.4 cm. The patient was diagnosed with left orbital cellulitis complicated with intracranial abscess.

The patient was admitted under a multidisciplinary team of ophthalmology, neurosurgery, neurology, and infectious diseases. He was referred to the dental department and otorhinolaryngology (ENT) department to look for the primary source of infection. There was generalized periodontitis, and dental extraction of three teeth was performed. Bedside nasal endoscopy was performed and thick pus discharge was found at the middle meatus, with erythematous and inflamed nasal mucosa. There was no fungal debris, necrotizing or crusting tissue. The pus discharge was sent for culture and revealed a mixed growth of microorganisms.

The patient was treated with intravenous ceftriaxone and metronidazole for five days. However, there was no clinical improvement. He had persistent temperature spikes up to 40 degrees Celsius and started to develop signs and symptoms of meningism, including neck stiffness, nausea, and photophobia in addition to headache. The right eye began to show signs of pre-septal cellulitis. An MRI of the brain and orbit was performed and it revealed bifrontal subdural collections, measuring 0.6 cm in maximum depth. Given the size of the lesion, surgical intervention was not recommended. The intravenous antibiotics were escalated to meropenem.

Due to the worsening condition, an anterior functional endoscopic sinus surgery was performed by the ENT team. There was minimal pus found at the anterior wall of the maxillary sinus, but otherwise, no fungal ball, eschar, or necrotic tissue was seen. All cultures taken intraoperatively were negative. A lumbar puncture was performed by the neurology team, and cerebrospinal fluid (CSF) culture and sensitivity result demonstrated *Peribacillus* growth which was sensitive to meropenem.

**Figure 2:**
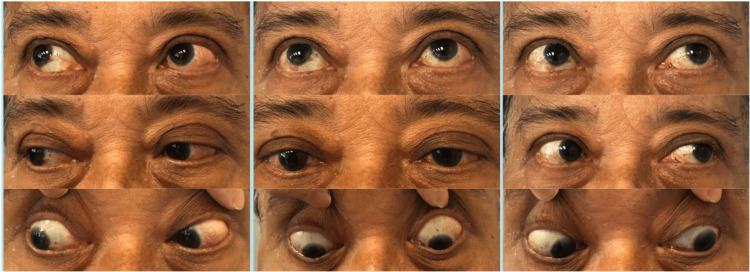
Photos taken six weeks after systemic antibiotic therapy. Ocular examination showed improved eye movement and reduced conjunctival chemosis.

The patient initially responded well to intravenous meropenem, with resolved conjunctival chemosis and improved extraocular movement (Figure 2). The vision of the left eye improved from 6/60 to 6/12. Intraocular pressure of the left eye was 12, controlled with two topical antiglaucoma.

**Figure 3:**
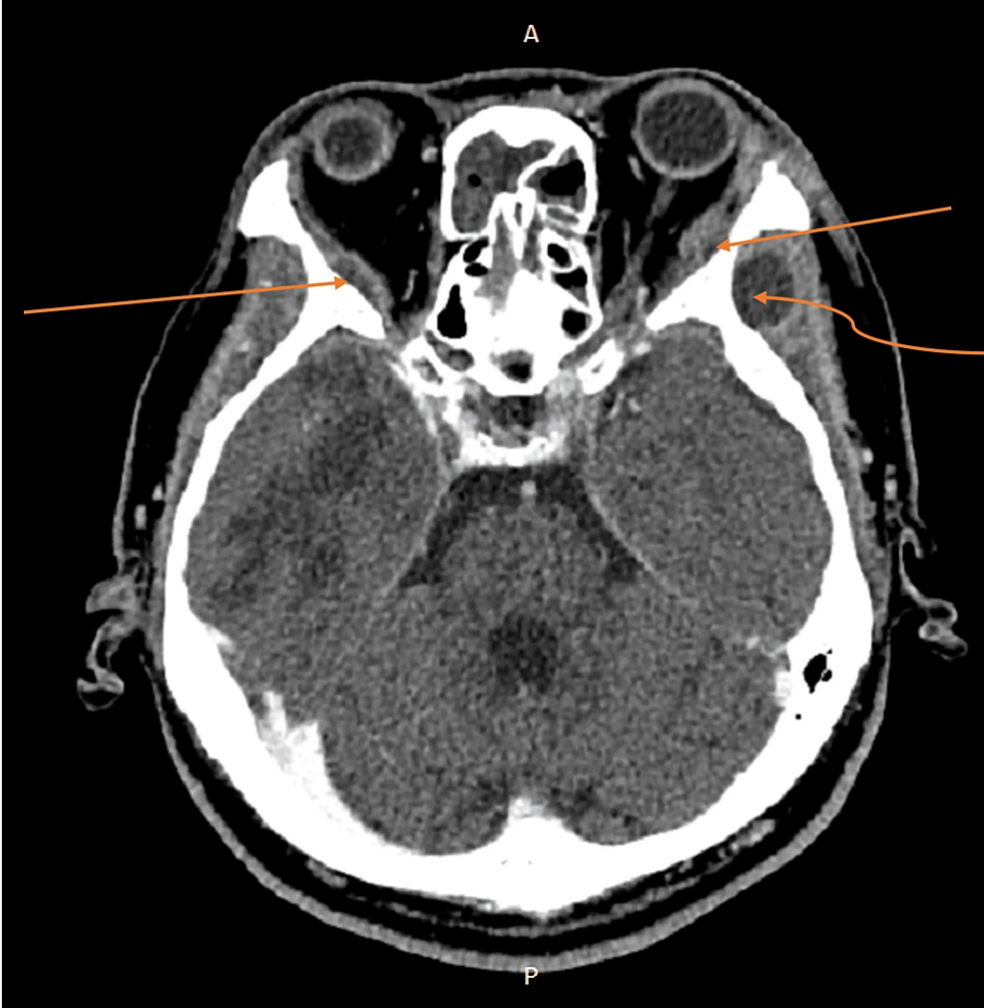
Repeat CECT six weeks after systemic antibiotic therapy shows the development of focal rim enhancing collections at the left lateral extraconal space measuring 0.6 cm x 1.6 cm and at the right lateral extraconal space measuring 0.5 cm x 1.6 cm (straight arrow). Also seen is the development of focal rim enhancing collection at the left temporalis measuring 1.6 cm x 1.3 cm (curved arrow). CECT: Contrast-enhanced computed tomography

**Figure 4:**
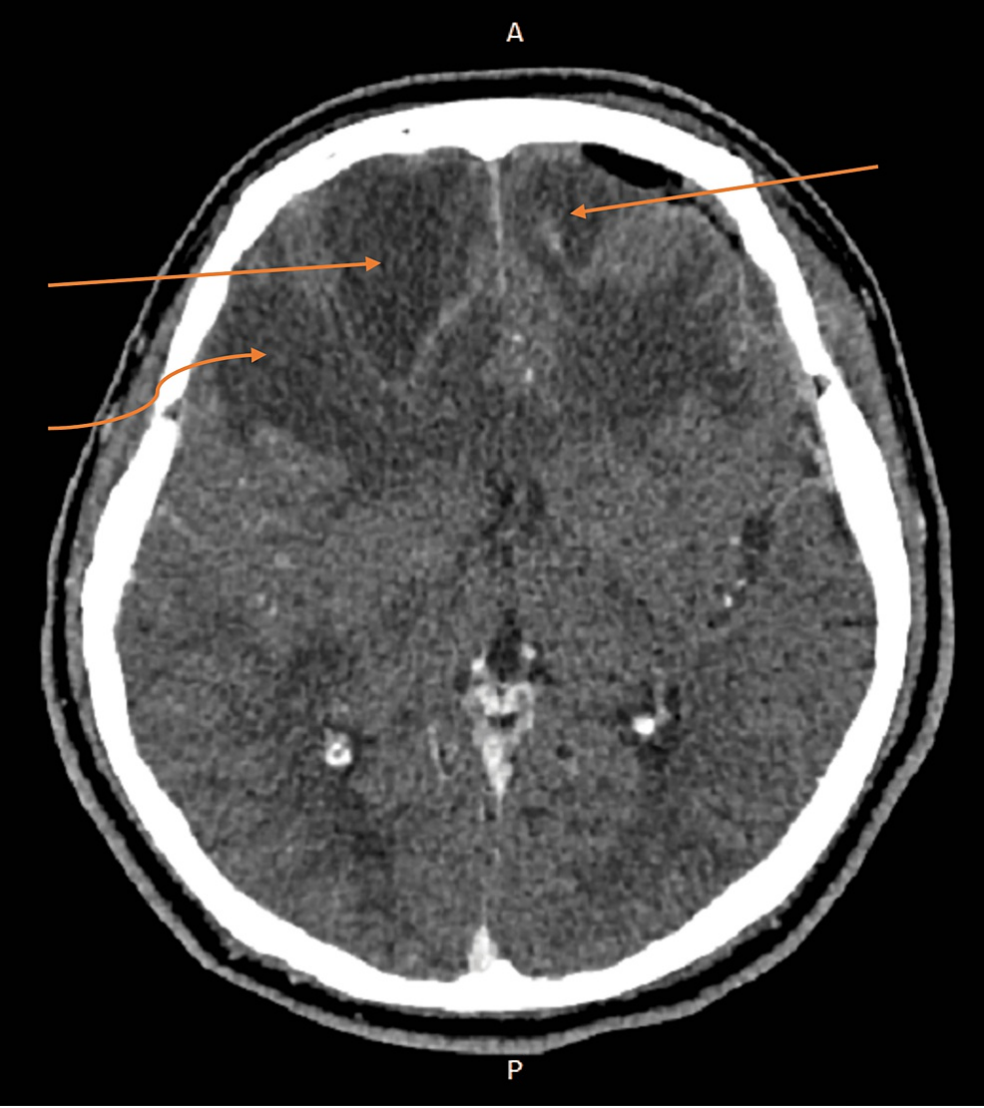
Repeat CECT six weeks after systemic antibiotic therapy shows ill-defined hypodense lesions within the bilateral frontal region with the right frontal lesion measuring 3.9 cm x 3.2 cm and the left frontal lesion measuring 2.0 cm x 2.2 cm (straight arrows). Also seen is an ill-defined hypodense lesion within the right temporal lobe measuring 2.8 cm x 2.4 cm (curved arrow). CECT: Contrast-enhanced computed tomography

After consulting with the infectious disease specialist, the choice of antibiotics was changed to intravenous ceftriaxone with a six-week duration. However, after completion of intravenous ceftriaxone, the patient’s condition deteriorated, with personality and behavioral changes, altered level of consciousness, and aphasia. There was no ocular sign or symptom. Repeated CECT brain revealed worsening bifrontal intracranial abscesses, with newly developed right temporal and left lateral extraconal hypodense lesions, suggestive of septic emboli (Figures 3-4). Repeated blood cultures and lumbar puncture were negative. The CECT scan of the thorax, abdomen, and pelvis demonstrated no foci of infection. The patient subsequently died eight weeks after the initial onset of presentation.

## Discussion

The odontogenic origin of orbital cellulitis is rare. The commonest predisposing cause of orbital cellulitis is sinus disease, followed by ocular trauma, ocular foreign bodies, and ocular surgery [3-5]. In a study of 214 patients with orbital cellulitis in the Middle East, dental infection was identified as the primary source in six (2.7%) of the cases [4]. However, odontogenic orbital cellulitis is commonly associated with sight- and life-threatening complications [6,7]. In our case, the patient had odontogenic orbital cellulitis complicated by intracranial abscess formation, resulting in mortality.

The commonest route of spread of odontogenic infection is via the maxillary sinus into the inferior orbit, through the inferior orbital fissure, or an orbital floor defect [6]. Other routes include dissemination via the pterygopalatine regions, the canine fossa, or retrograde spread via the ophthalmic vein [6]. A review found that odontogenic orbital cellulitis is commonly associated with subperiosteal or orbital abscess formation, as demonstrated in our case [6]. More than half the reported cases required surgical drainage [6]. It is important to have a high index of suspicion, as some patients may not have any recent history of dental surgery or complaints [8].

In addition to the rare primary source of the orbital cellulitis, our patient’s condition was complicated by meningitis and intracranial abscess formation. However, no clinically significant organism was found from the culture of CSF or sinus aspirate. A review by Tsirouki et al. found that the commonest causative organisms of orbital cellulitis were Staphylococcus aureus and Streptococcus species, while mixed aerobic and anaerobic bacteria were the main causative organisms in odontogenic orbital cellulitis [3]. In our case, the Peribacillus species was found in the CSF culture. This soil-related organism is generally considered nonpathogenic and can be a contaminant [9]. However, it may also have a role in bacterial virulence by producing biofilms and secreting proteases [9].

Identification of the causative organisms associated with orbital cellulitis is difficult due to normal flora contamination, prior antibiotic therapy, and mixed infections [10]. Cultures from sinus aspirates and orbital abscesses have the highest reliability, followed by cultures from nasal swabs, throat swabs, and ocular secretions [3]. Blood cultures are often negative [3]. A meta-analysis found that only 24% of CSF cultures in cases of intracranial abscesses have a positive yield [11]. In our case, anterior functional endoscopic sinus surgery with sinus aspirate collection was done after a week of intensive antibiotic therapy. The prior antibiotic therapy and the long interval between antibiotic exposure and sample collection may affect the bacterial yield and identification [12].

Brain imaging is important in the diagnosis and management of intracranial abscesses. Comparison of serial CECT scans in addition to clinical judgment is useful as a tool for monitoring treatment response and disease progression. In this case, the improvement in the ocular symptoms might mislead the clinical judgment. However, careful examination revealed the development of behavioral changes and an altered mental status in this patient. Serial imaging of the brain revealed worsening intracranial abscesses. Hence, a high index of suspicion is necessary as the neurological presentation of an intracranial abscess could be subtle, and brain imaging is recommended [13].

Early, aggressive intervention is fundamental in the treatment of orbital cellulitis. Immediate initiation of broad-spectrum antibiotic therapy is necessary after obtaining cultures. The use of third-generation cephalosporins such as ceftriaxone with flucloxacillin is recommended, as it covers the usual causative bacteria [3]. In cases of intracranial extension, a multidisciplinary approach of ENT, neurosurgeons, ophthalmologists, and infectious disease specialists is paramount. The use of wide-spectrum antibiotics with good central nervous system (CNS) penetration is important as brain abscesses could be polymicrobial [3,13,14].

## Conclusions

This report demonstrated a case of fatal orbital cellulitis, with the probable primary source of infection being odontogenic. It was complicated by the formation of intracranial abscesses. The patient’s condition worsened despite extensive investigations and the immediate initiation of empirical antibiotics on presentation. A high index of suspicion to investigate the primary source of infection and identify complications is important in cases of orbital cellulitis. Specimen collection from the orbital abscess and sinus aspirate is recommended. Early consideration of surgical management might be beneficial in cases of orbital cellulitis with intracranial abscess formation.
